# Red Wine Grape Pomace Restores Gut Barrier Function and Improves Survival in Diet-Induced Ischemic Heart Disease

**DOI:** 10.3390/antiox14050574

**Published:** 2025-05-10

**Authors:** Katherine Rivera, Leticia González, Laura Parra, Juan E. Oyarzún, Alina Concepción-Alvarez, Adriano Costa de Camargo, Raquel Bridi, Attilio Rigotti, Marcelo E. Andia

**Affiliations:** 1 Doctoral Program in Medical Sciences, Faculty of Medicine, Pontificia Universidad Católica de Chile, Santiago 8331010, Chile; ksrivera@uc.cl (K.R.);; 2 Radiology Department and Biomedical Imaging Center, School of Medicine, Pontificia Universidad Católica de Chile, Santiago 7820436, Chile; 3 Millennium Institute for Intelligent Healthcare Engineering iHEALTH, Santiago 7820436, Chile; 4 Nutrition and Food Technology Institute, University of Chile, Santiago 7830490, Chile; 5 Departamento de Química Farmacológica y Toxicológica, Facultad de Ciencias Químicas y Farmacéuticas, Universidad de Chile, Santiago 8380494, Chile; 6 Centro de Nutrición Molecular y Enfermedades Crónicas, Escuela de Medicina, Pontificia Universidad Católica de Chile, Santiago 8331010, Chile

**Keywords:** phenolic compounds, red wine grape pomace, endotoxemia, gut permeability, ischemic heart disease, gut–heart axis

## Abstract

Red wine grape pomace (RWGP), a winemaking by-product rich in phenolics, flavonoids, and dietary fiber, has shown promise in mitigating cardiovascular disease (CVD), however, its mechanisms of action remain incompletely understood. This study comprehensively profiled the phenolic composition of RWGP—including free, esterified, etherified, and insoluble-bound fractions—and evaluated the effects of RWGP dietary supplementation on gut barrier integrity, inflammation, oxidative stress, and survival in SR-B1^−/−^ApoE-R61^h/h^ mice, a model of diet-induced lethal ischemic heart disease. RWGP supplementation significantly improved survival rates and restored gut barrier function, as evidenced by lower plasma FITC-dextran and LPS levels, increased circulating ZO-1 levels, and reduced histopathological colon damage. In addition, RWGP reduced pro-inflammatory cytokines (IL-1β) and showed a trend toward attenuating systemic oxidative stress (TBARS). Analysis of phenolic compounds indicated a significant presence of insoluble-bound phenolics. Nevertheless, the beneficial effects observed are likely attributable to the synergistic actions of RWGP’s complex phytochemical and fiber composition. These results highlight RWGP’s potential as a sustainable, gut-targeted functional food ingredient for CVD prevention and management.

## 1. Introduction

Ischemic heart disease remains the leading cause of global mortality, with diet playing a pivotal role in modulating associated risk factors [1]. Although traditional pharmacological treatments, such as statins, have been effective in improving cardiovascular outcomes, there is increasing interest in using food-based approaches as complementary or preventative strategies that target the fundamental pathophysiological mechanisms involved in disease [2].

Emergent evidence underscores the role of the gut–heart axis, a bidirectional connection between intestinal integrity and cardiovascular function, in the pathogenesis of cardiovascular diseases (CVD) [3,4]. A compromised intestinal barrier, often exacerbated by Western diet patterns, increases intestinal permeability, facilitating the translocation of microbial products such as lipopolysaccharide (LPS) into the systemic circulation—a process known as metabolic endotoxemia [5,6]. This phenomenon triggers low-grade systemic inflammation, a key contributor to atherosclerosis progression, myocardial injury, and adverse cardiac remodeling [7,8,9,10]. Interventions that restore gut barrier function may thus offer cardiometabolic benefits beyond those conferred by lipid-lowering therapies.

Red wine grape pomace (RWGP)—a by-product of the winemaking process primary composed of grape skins, stems, and seeds (*Vitis vinifera* L.)—has garnered interest due to its rich and complex matrix of bioactive constituents, including soluble and insoluble-bound phenolics, flavonoids, and dietary fiber [11,12,13,14]. Traditionally considered agro-industrial waste, RWGP has demonstrated beneficial effects against key drivers of CVD progression, including endothelial function, oxidative stress, and atherosclerosis burden [15,16,17,18,19]. Previous research showed that RWGP supplementation enhanced survival in a mouse model of diet-induced lethal ischemic heart disease, which was not replicated with fiber supplementation alone, indicating that RWGP’s benefits likely arise from the synergistic action of its diverse phytochemicals and fiber matrix [19]. Although considerable evidence supports the cardioprotective effects of RWGP, the specific mechanisms linking these benefits to gut barrier function remain unclear. Furthermore, while earlier research focused on individual components, especially soluble phenolics, more recent findings emphasize the significance of the intricate interactions among RWGP’s constituents as they move through the gastrointestinal tract [20].

This study provides a comprehensive analysis of the phenolic compounds in RWGP, including free, esterified, etherified, and insoluble-bound forms. Furthermore, it investigates the impact of RWGP on gut permeability, systemic oxidative stress, inflammation, and survival using a murine model of diet-induced lethal ischemic heart disease. By examining the combined effects of RWGP’s bioactive and structural elements, this research offers new perspectives on its capacity to protect the intestinal barrier and its potential as a sustainable dietary approach for improving cardiometabolic health.

## 2. Materials and Methods

### 2.1. RWGP Material

RWGP flour was previously obtained from late-harvest red wine grapes (Cabernet Sauvignon, vintage 2011, Maipo Valley, Chile) [17]. Briefly, the frozen RWGP was thawed at room temperature and dried in a forced air dryer at 60 °C until the moisture reached less than 12%. The dried pomace was powdered, packaged, and stored at −20 °C until use.

### 2.2. Phenolic Compound Fractionation, Identification, and Quantification

**Figure 1:**
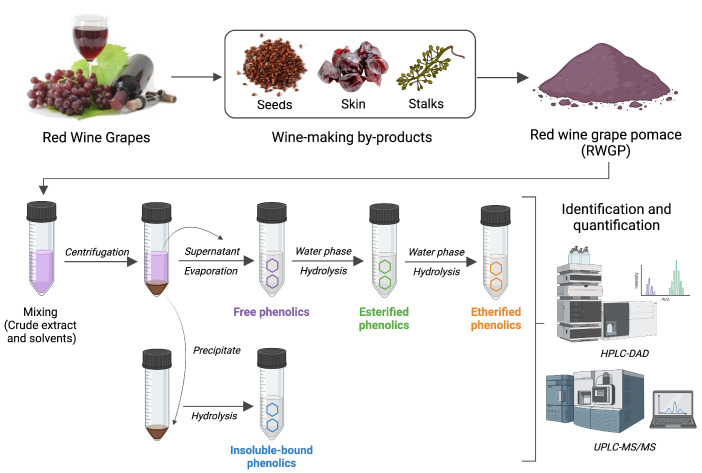
Main steps for the extraction of soluble (free, esterified, etherified) and insoluble-bound phenolic compounds from RWGP. Created in BioRender.com.

Phenolic compounds in RWGP were separated into four major fractions—free, esterified, etherified, and insoluble-bound—using a sequential extraction and hydrolysis protocol based on established methods with minor modifications (Figure 1) [21,22,23]. These fractions are distinguished by their chemical associations with the plant matrix: free phenolics are soluble and unbound, esterified phenolics are linked via ester bonds to cell wall polysaccharides (e.g., arabinoxylans, pectins), etherified phenolics are covalently linked via ether bonds to structural polymers such as lignin or cellulose, and insoluble-bound phenolics are tightly bound to insoluble dietary fiber components via ester or ether linkages and not extractable without strong alkaline hydrolysis [24].

#### 2.2.1. Extraction of Soluble Free, Esterified, and Etherified Phenolics

Five grams of dry RWGP flour was treated with 150 mL of acetone/water (70:30, *v*/*v*) extraction solution and subjected to sonication for 20 min at room temperature. The mixture was centrifuged at 5000× *g* for 5 min, and the supernatant was decanted and retained. The residue was extracted twice more under the same conditions. The combined supernatants were evaporated under vacuum at 40 °C using a rotary evaporator to obtain a free-acetone aqueous extract, which was used for the extraction of free, esterified, and etherified phenolics [21,22]. Briefly, the aqueous phase was acidified to pH 2.0 using 6 M HCl and subjected to five liquid–liquid extractions with ethyl acetate (1:1, *v*/*v*). The combined ethyl acetate extracts were evaporated at 40 °C to obtain a solid residue, which was dissolved in 5 mL of methanol and stored at −20 °C for free phenolic analysis. The remaining aqueous layer was hydrolyzed with 4 M NaOH for 4 h at room temperature to release esterified phenolics. After acidification to pH 2 with 6 M HCl, phenolic compounds were extracted with ethyl acetate as described above. The combined ethyl acetate extracts were evaporated to dryness under vacuum at 40 °C, and the residue was dissolved in 5 mL of methanol for esterified phenolic analysis. For etherified phenolic extraction, the remaining aqueous phase was acidified with 6 M HCl and incubated at 90 °C for 45 min to cleave glycosidic bounds. After cooling, the released phenolics were extracted five times with ethyl acetate (1:1, *v*/*v*). The combined ethyl acetate extracts were evaporated to dryness under vacuum, and the residue was dissolved in 5 mL of methanol for etherified analysis [23]. All extracts were protected from light by covering containers with aluminum foil.

#### 2.2.2. Extraction of Insoluble-Bound Phenolics

The solid residue obtained after acetone/water extraction was dried at 40 °C for 24 h and subjected to alkaline hydrolysis with 2 M NaOH for 4 h at room temperature under nitrogen. The solution was acidified to pH 2.0 with 6 M HCl, and insoluble-bound phenolics were extracted five times with ethyl acetate (1:1, *v*/*v*). The combined organic phases were evaporated to dryness under vacuum, and the residue was dissolved in 5 mL of methanol for analysis. All extracts were stored at −20 ºC to prevent degradation [23,25].

#### 2.2.3. Determination of Total Phenolic and Flavonoid Content

Total phenolic content was determined using the Folin–Ciocalteu method according to Singleton et al., with modifications described by Bridi et al. [26,27]. This independent measurement was included in addition to the summation of fractionated phenolics (free, esterified, etherified, and insoluble-bound) to provide a reference value for comparison and to account for possible phenolic losses during sequential extraction, thereby improving the reliability and consistency of total phenolic estimates. Briefly, diluted phenolic extracts (1:10, *v*/*v*) were mixed with Folin–Ciocalteu reagent and sodium carbonate (${\mathrm{Na}}_{2}$${\mathrm{CO}}_{3}$, 75 g/L). This mixture was incubated at 37 °C for 30 min, and absorbance was measured at 756 nm using a microplate reader. A gallic acid calibration curve was used for quantification, and results were expressed as milligrams of gallic acid equivalents (GAE) per 100 g of sample (mg GAE/100 g). Total flavonoid content was determined using the ${\mathrm{AlCl}}_{3}$ colorimetric method, with ${\mathrm{NaNO}}_{2}$ added in a basic medium to enhance reactivity in complex matrices [28]. Absorbance was measured at 510 nm, and quantification was performed using a quercetin calibration curve. Results were expressed in milligrams of quercetin equivalents (QE) per 100 g of sample (mg QE/100 g). All measurements were performed in triplicate, and results are reported as means ± standard deviations (SD).

#### 2.2.4. Characterization of Phenolic Compounds by HPLC-DAD

Phenolic compounds in the different fractions were identified using high-performance liquid chromatography coupled to a diode array detector (HPLC-DAD) (Hitachi Chrommaster 5000 series, Tokyo, Japan). The system was equipped with an autosampler and photodiode array detector, controlled by Chromaster System Manager v1.2 software. A Purospher STAR RP-18 end-capped column (250 mm × 4.6 mm, Merck, Darmstadt, Germany) with a guard column of the same type was used. Samples were injected in triplicate, and separation was achieved using a mobile phase gradient composed of acetonitrile and acidified water (0.1 % formic acid) at a flow rate of 1.0 mL/min at 35 °C (Table 1). Absorbance was monitored from 210 to 550 nm, and chromatograms were integrated at 290 nm. Phenolic compounds identification was performed by comparison of the retention times exhibited by standards and UV–visible spectra. Quantification was performed using calibration curves for each compound with range between 5 and 100 μg/mL, and the results were expressed in mg/100 g sample. Additionally, minor compounds were identified by ultra-performance liquid chromatography coupled with tandem mass spectrometry (UPLC-MS/MS) using an ABSciex triple Quad 4500 mass spectrometer combined with an Eksigent Ekspert Ultra LC100 and an LC100-XL autosampler system (ABSciex, Concord, ON, Canada). Electrospray was used in negative mode. Chromatographic separation was carried out as published elsewhere [22].

### 2.3. Animal Model and Diets

SR-B1^−/−^ApoE-R61^h/h^ mice, originally obtained from Dr. Monty Krieger (Massachusetts Institute of Technology (MIT), Cambridge, MA, USA), were maintained in the animal facility of the School of Medicine at Pontificia Universidad Católica de Chile. Mice were housed under controlled light (12 h light/dark cycle), temperature (22 ± 2 °C), and humidity (50 ± 10%) conditions with free access to water and control diet (Prolab RMH 3000; PMI Feeds Inc., Brentwood, CA, USA), containing 5% fat. The atherogenic diet (Cocoa Test Diet 57BB, St Louis, MO, USA) containing 15.5% fat, 1.25% cholesterol, and 0.5% cholic acid, was used to induce the ischemic heart disease over three weeks of dietary intervention. Briefly, at 8–10 weeks of age, SR-B1^−/−^ApoE-R61^h/h^ male mice were randomized into four dietary treatment groups: (1) 100% control diet (CD); (2) 80% atherogenic diet + 20% control diet (HFCD-CD), (2) 80% atherogenic diet + 20% control diet + 20 mg/kg atorvastatin (HFCD-ST), and (4) 80% atherogenic diet + 20% RWGP flour (HFCD-RWGP). This dose of RWGP supplementation was based on our previous dose–response optimization studies with the same murine models of atherosclerosis [19]. Two sets of animals were used: one set (n = 8–10) for survival monitoring and another set (n = 5–8) for biochemical and histological determinations. Body weight and food intake were recorded twice weekly. This study was conducted in accordance with institutional guidelines from the Guide for the Care and Use of Laboratory Animals published by the US National Institutes of Health.

#### 2.3.1. Survival Analysis

The survival rate was determined by tabulating the time to death or euthanasia for each mouse and plotting Kaplan–Meier survival. All animals were visually inspected daily to assess their health status. Criteria for euthanasia were based on institutional guidelines and included poor general appearance, abnormal behavior (e.g., ruffled fur, abnormal gait), reduced food and water intake, a body weight loss of > 20%, or reduced activity lasting > 48 h. Animals reaching these humane endpoints were euthanized, and both spontaneous deaths and euthanized animals were included in the survival curves without distinction. Survival curves were compared using the log-rank test, with *p* < 0.05 considered statistically significant.

#### 2.3.2. Intestinal Permeability Assay

An in vivo intestinal permeability assay was performed to assess gut epithelial barrier function, as previously described [29]. After 3 weeks of dietary intervention, mice were fasted for 4 h to ensure consistent absorption of FITC-dextran and minimize variability due to food intake. Mice were orally gavaged with 4 kDa FITC-dextran (600 mg/kg, Sigma-Aldrich, St. Louis, MO, USA), prepared from an 80 mg/mL stock solution. One hour after gavage, blood was collected by cardiac puncture using citrate as an anticoagulant and centrifuged at 4400 xrpm for 15 min at 4 ºC. Plasma was diluted 1:2 in PBS and protected from light until analysis. Fluorescence was quantified using a microplate reader at an excitation wavelength of 480 nm and emission wavelength of 535 nm. Concentrations of FITC-dextran were calculated using a standard curve prepared from serial dilution of FITC-dextran.

#### 2.3.3. Sample Collection

After 3 weeks of dietary intervention, the mice were weighed and euthanized by intra-peritoneal injection of a mixture of ketamine:xylazine mixture (150:10 mg/kg) to ensure rapid and humane euthanasia. Blood samples (approximately 0.5–1 mL per mouse) were collected by cardiac puncture using citrate as an anticoagulant and immediately centrifuged at 44,000 xrpm for 15 min at 4 ºC to separate plasma from cells. The colon was removed, and its length (from the cecum to the rectum) was measured and photographed. Segments from the proximal colon were gently rinsed with 0.9% NaCl to remove luminal contents, fixed in 10% buffered neutral formalin solution for 24 h, and embedded in paraffin for histological evaluation. Tissue sections were stained with hematoxylin and eosin (HE), Masson’s Trichrome, and Picrosirius Red to assess epithelial integrity and intestinal fibrosis.

#### 2.3.4. Biomarkers of Intestinal Permeability

Plasma zonulin (ZO-1) concentrations were measured using a commercial by enzyme-linked immunosorbent assay (ELISA) kit (Zonulin ELISA kit, CUSABIO Technology LLC, Houston, TX, USA) with a sensitivity limit of 0.156 ng/mL and a detection range of 0.625–40 ng/mL. Systemic LPS levels were determined using a chromogenic endotoxin quantification kit (Pierce™ Chromogenic Endotoxin A39553, Thermo Fisher Scientific, MA, USA) according to the manufacturer’s instructions. Briefly, plasma samples were diluted 1:10 with pyrogen-free water and incubated at 70 °C for 15 min to inactivate inhibitors. Aliquots of 50 μL of each standard and sample were transferred in triplicate to a 96-well flat-bottomed microplate and incubated at 37 °C. Following the addition of 50 μL Limulus Amebocyte Lysate (LAL) reagent per well, kinetic color development at 405 nm was recorded. A standard curve was generated using a serial dilution of LPS standard, and endotoxin content in the individual samples was calculated.

#### 2.3.5. Oxidative Stress and Inflammatory Markers

Plasma levels of lipid peroxidation and pro-inflammatory cytokines were assessed to evaluate systemic oxidative stress and inflammation. TBARS (thiobarbituric acid reactive substances) were measured using a colorimetric assay (Cayman Chemical, Ann Arbor, MI, USA; Cat. No. 10009055) following the manufacturer’s instructions. Absorbance was read at 532 nm, and results were expressed as malondialdehyde (MDA) equivalents (μM). For cytokine quantification, plasma concentrations of interleukin-1 (IL-1β) and tumor necrosis factor-alpha (TNF-α) were determined using commercial ELISA kits (R&D Systems, Minneapolis, MN, USA; Cat. No. MLB00C and MTA00B, respectively). Samples were run in duplicate, and absorbance was measured at 450 nm with wavelength correction at 540 nm using a microplate reader. Cytokine concentrations were calculated from standard curves and expressed in pg/mL.

#### 2.3.6. Colon Histopathology Analysis

Histomorphometric changes in the colon were evaluated by measuring crypt depth, mucosal thickness, and tunica muscularis thickness. For each parameter, different sections were taken from different areas of the colon, and measurements were performed at 20× magnification using image analysis software Fiji [30]. All data for each parameter were averaged per mouse, with a minimum of four measurements for each parameter per mouse. The severity of inflammation and damage in the colon tissue was evaluated using histopathological scoring considering the following parameters: inflammatory infiltration, damage to mucosal architecture, submucosal edema, and collagen deposition. A score (0–4) was assigned to each parameter, and then summed to obtain an overall index of tissue damage.

#### 2.3.7. Statistical Analysis

Data from phenolic compounds analysis are expressed as the mean ± standard deviation (SD). Statistical comparisons between three or more groups were performed using one-way ANOVA followed by Tukey’s post hoc test. In contrast, in vivo data are expressed as the median ± range. Statistical comparisons between groups were performed using the Kruskal–Wallis test followed by Dunn’s post hoc test. Significance was accepted at *p* < 0.05. All statistical analyses were performed using GraphPad Prism software (version 10.1, GraphPad Software, San Diego, CA, USA).

## 3. Results

### 3.1. Total Phenolic and Flavonoid Content

The total phenolic and flavonoid content of RWGP flour varied significantly across its soluble free, esterified, etherified, and insoluble-bound fractions (Table 2). The insoluble-bound fraction exhibited the highest total phenolics (830 ± 42 mg GAE/100 g) and flavonoids content (656 ± 56 mg QE/100 g), accounting for 49.28% and 48.38% of total phenolic and flavonoid content, respectively (*p* < 0.05 vs. other fractions).

The free fraction also contained significant levels of phenolics (509 ± 43 mg GAE/100 g) and flavonoids (535 ± 6.7 mg QE/100 g), while the esterified and etherified fractions contributed a lower amount (270 ± 20 mg GAE/100 g and 75 ± 9 mg GAE/100 g for total phenolics; 116 ± 6.9 mg QE/100 g and 49 ± 5.9 mg QE/100 for flavonoids). Cumulatively, RWGP flour contained 1684 mg GAE/100 g of total phenolics and 1356 mg QE/100 g of total flavonoids, underscoring its potential as a functional food ingredient rich in bioactive compounds.

### 3.2. Identification and Quantification of Phenolic Compounds

Phenolic acids, monomeric flavanols, and dimeric flavanols were identified and quantified in RWGP, with significant differences in their distribution across the four fractions (Table 3). Gallic acid was the most abundant phenolic acid, predominantly found in the insoluble-bound fraction (72.38 ± 0.03 mg/100 g), followed by the esterified fraction (17.05 ± 0.22 mg/100 g). p-Coumaric acid was concentrated in the esterified (21.20 ± 0.20 mg/100 g) and insoluble-bound (34.91 ± 0.57 mg/100 g) fractions. Catechin and epicatechin were the most abundant flavonoids. Catechin was highest in the insoluble-bound fraction (26.65 ± 1.79 mg/100 g), whereas epicatechin was prominent in the free fraction (40.75 ± 0.68 mg/100 g). Procyanidin B2, a polymeric flavanol, was significantly enriched in the insoluble-bound fraction (7.19 ± 0.68 mg/100 g), compared to the esterified fraction (1.16 ± 1.01 mg/100 g). Kaempferol was exclusively detected in the free fraction (2.16 ± 0.07 mg/100 g). Minor phenolic compounds such as syringic acid, ferulic acid, caffeic acid, chlorogenic acid, and quercetin were detected in all fractions by UPLC-ESI-MS/MS, whereas rutin was found only in the free form. Pinocembrin (free, esterified, and insoluble-bound) and apigenin (free and esterified) were also detected as a minor compound.

### 3.3. RWGP Supplementation Improves Survival and Gut Integrity

#### 3.3.1. Survival Analysis and Body Weight

SR-B1^−/−^ApoE-R61^h/h^ mice fed the high-fat, high-cholesterol, cholic acid-containing diet (HFCD-CD) exhibited several morbidities, evidenced by messy hair, diarrhea, and significant body weight loss (−3.90 g, *p* = 0.0065), compared to control mice (CD) exhibiting normal growth, active behavior, stable body weight, and normal stool (Table 4).

**Figure 2:**
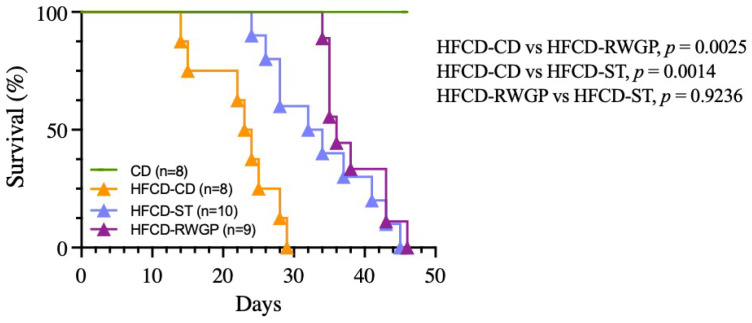
Effect of RWGP supplementation on survival of SR-B1^−/−^ApoE-R61^h/h^ mice. Kaplan–Meier curves based on log-rank test for CD, HFCD-CD, HFCD-ST, and HFCD-RWGP groups.

The diet-induced heart disease progression was associated with premature death, with a median survival of only 23.5 days (Figure 2). In contrast, RWGP supplementation (HFCD-RWGP) significantly extended median survival to 36 days (*p* = 0.0025 vs. HFCD-CD), an effect comparable to the statin-treated group (HFCD-ST: 33 days, *p* = 0.9236 vs. HFCD-RWGP) (Figure 2). Despite higher fat intake, RWGP-supplemented mice attenuated weight loss induced by the atherogenic diet (final body weight: 27.20 g vs. HFCD-CD: 23.80 g; Table 4). These findings suggest RWGP exerts a protective effect beyond metabolic regulation.

#### 3.3.2. RWGP Reduces Gut Permeability and Endotoxemia

**Figure 3:**
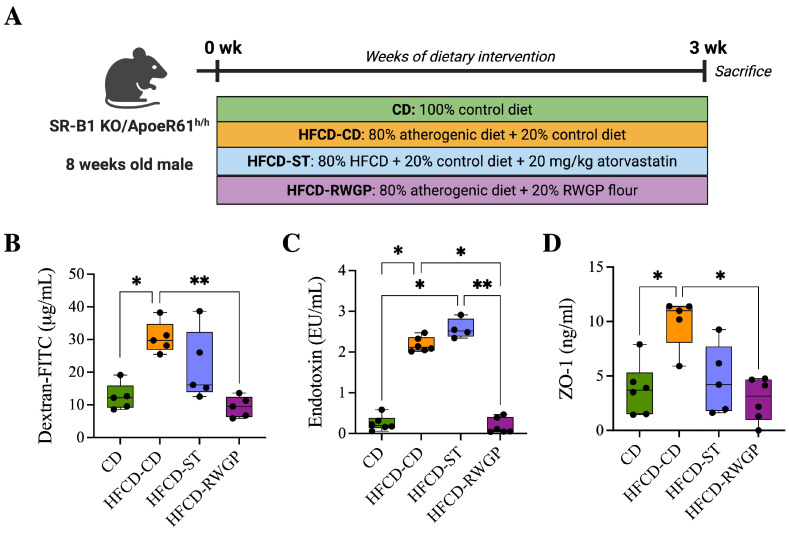
Effect of RWGP supplementation on gut permeability function of SR-B1^−/−^ApoE-R61^h/h^ mice. (**A**) In vivo experimental design. (**B**) Quantification of dextran-FITC in plasma (μg/mL). (**C**) Plasma endotoxin concentration (EU/mL). (**D**) Plasma ZO-1 levels (ng/mL). Kruskal–Wallis test, followed by Dunn’s test for multiple comparisons. * *p* < 0.05, ** *p* < 0.01.

RWGP’s phenolic composition, particularly its high insoluble-bound phenolic content, suggests that RWGP’s bioactivity may extend beyond direct antioxidant effects, influencing intestinal barrier function. To assess that, we analyze the effects of RWGP dietary supplementation on intestinal permeability markers in our model after 3 weeks of dietary intervention (Figure 3A). Plasma FITC-dextran, a direct marker of intestinal permeability, varied significantly between the experimental groups, with the HFCD-CD group showing elevated levels compared to the CD group (29.71 μg/mL vs. 12.18 μg/mL, *p* = 0.420), indicating increased intestinal permeability. RWGP supplementation significantly reduced FITC-dextran levels compared to HFCD-CD (9.49 μg/mL; *p* = 0067), to near-normal levels comparable to the CD group (*p* > 0.05, Figure 3B). Atherogenic diet intake was also correlated with elevated plasma LPS levels compared CD group (1.09 EU/mL, *p* = 0.0197), indicating increased bacterial translocation. RWGP supplementation lowered LPS levels to 0.56 EU/mL (*p* = 0.0351 vs. HFCD-CD), comparable to control diet (CD: 0.20 EU/mL, *p* > 0.05), indicating a lower translocation of bacterial LPS into systemic circulation (Figure 3C). Plasma ZO-1 levels, indicative of tight junction protein degradation, were significantly higher in the HFCD-CD group (10.99 ng/mL) compared to the CD group (3.71 ng/mL, *p* = 0.0432), indicating tight junction degradation. RWGP supplementation reduced ZO-1 levels (3.15 ng/mL vs. HFCD-CD: 10.99 ng/mL; *p* = 0.0196), suggesting preserved tight junction integrity and enhanced gut barrier function (Figure 3D). Statin-treated mice (HFCD-ST) showed partial protection, with intermediate FITC-dextran and ZO-1 values, suggesting a lesser impact on gut barrier function compared to RWGP.

#### 3.3.3. Effect of RWGP Supplementation on Oxidative Stress and Inflammatory Markers

**Figure 4:**
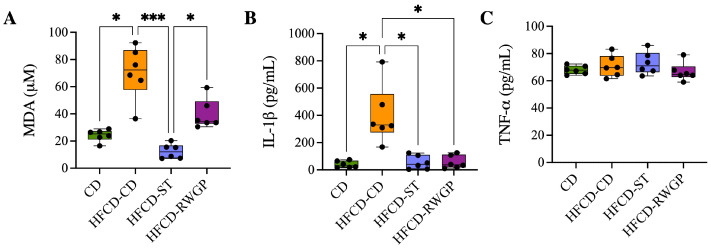
Effect of RWGP supplementation on oxidative stress and inflammatory markers. Plasma levels of (**A**) malondialdehyde (MDA), (**B**) interleukin-1β (IL-1β), and (**C**) tumor necrosis factor-alpha (TNF-α). Kruskal–Wallis test, followed by Dunn’s test for multiple comparisons. * *p* < 0.05, *** *p* < 0.001.

Systemic oxidative stress and inflammation were assessed by measuring plasma levels of malondialdehyde (MDA, as TBARS), IL-1β, and TNF-α (Figure 4). Mice fed an atherogenic diet (HFCD-CD) exhibited significantly elevated MDA levels compared to the CD group (72.25 μM vs. 25.17 μM, *p* = 0.0224), indicating increased lipid peroxidation (Figure 4A). The statin-treated group exhibited a significant reduction in MDA levels relative to HFCD-CD (12.06 μM, *p* = 0.0001), also indicating effective attenuation of oxidative stress. RWGP supplementation (HFCD-RWGP) did not significantly reduce MDA levels relative to the HFCD-CD group (72.25 μM vs. 34.33 μM, *p* > 0.05), though levels were slightly lower. Similarly, IL-1β levels were markedly elevated in HFCD-CD mice compared to CD group (329.4 pg/mL vs. 32.03 pg/mL, *p* = 0.0132) (Figure 4B). Both RWGP (37.37 pg/mL, *p* = 0.0374 vs. HFCD-CD) and statin treatment (41.37 pg/mL, *p* = 0.0151 vs. HFCD-CD) significantly reduced IL-1β levels, suggesting anti-inflammatory effects through modulation of this cytokine. No significant differences in plasma TNF-α levels were observed among groups (all *p* > 0.05) (Figure 4C).

#### 3.3.4. RWGP Mitigates Atherogenic Diet-Induced Colon Damage

**Figure 5:**
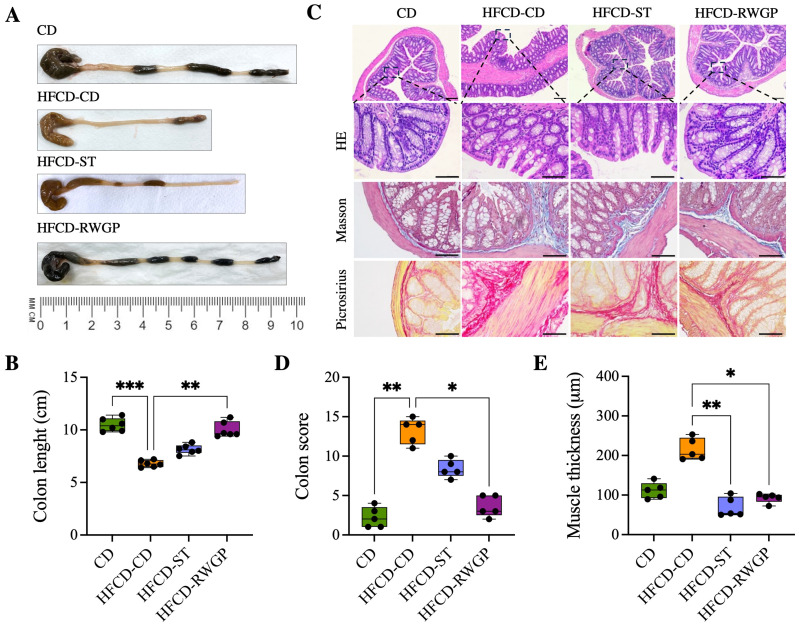
Effect of RWGP supplementation on gut integrity of SR-B1^−/−^ApoE-R61^h/h^ mice. (**A**) Representative image of the colon for each intervention. (**B**) Quantification of colon length (cm). (**C**) Histomorphology of colon. (**D**) Colon score. (**E**) Colon muscle thickness (μm). Scale bar represents 200 μm. Kruskal–Wallis test, followed by Dunn’s test for multiple comparisons. * *p* < 0.05, ** *p* < 0.01, *** *p* < 0.001.

Figure 5 shows the effect of RWGP supplementation on gut integrity (Figure 5). HFCD-CD mice showed marked colon shortening compared to CD (6.75 cm vs. 10.4 cm, *p* = 0.0003), indicative of chronic inflammation and fibrosis (Figure 5A). RWGP supplementation significantly reversed colon shortening (9.65 cm, *p* = 0.0056 vs. HFCD-CD), whereas the statin-treated group (HFCD-ST) did not show significant improvements (*p* > 0.05) (Figure 5A,B). Histopathological analysis with HE, Masson’s Trichrome, Picrosirius Red staining evidenced that CD mice exhibited normal colonic structure, with no edema, inflammation or fibrosis (Figure 5C). HFCD-CD mice showed severe inflammatory cell infiltration, mucosal damage, and submucosal fibrosis. Notably, RWGP supplementation significantly attenuated fibrosis and restored colonic architecture, with lower histological scores and decreased mucus layer thickness (*p* = 0.0175 vs. HFCD-CD) (Figure 5D,E). The statin-treated group exhibited partial protection, but showed persistent fibrosis and inflammation (*p* < 0.05 vs. HFCD-CD).

## 4. Discussion

Although RWGP—a winemaking by-product rich in phenolics, flavonoids, and dietary fiber—has previously demonstrated cardioprotective effects, including reduced mortality in diet-induced ischemic heart disease models, the mechanistic pathways linking these effects with intestinal barrier function remain underexplored. This work introduces novel endpoints focused on the gut, including intestinal permeability (FITC-dextran assay), systemic endotoxemia (plasma LPS), intestinal epithelial integrity (ZO-1 quantification), and histological scoring of colon morphology, to investigate the gut–heart axis in a unique model of diet-induced lethal ischemic heart disease. We showed that RWGP significantly improves the function of the gut barrier, reduces systemic endotoxemia and inflammation, and enhances survival in a murine model of diet-induced ischemic heart disease. Rather than attributing these effects to a single component, our findings reinforce the hypothesis that RWGP’s benefits arise from synergistic interactions within its complex phytochemical fiber matrix, collectively contributing to intestinal and cardiovascular protection. These integrative analyses provide new mechanistic insights into the role of the complex phytochemical fiber matrix of RWGP in preserving gut integrity and modulating systemic inflammation.

RWGP’s high content of insoluble-bound phenolics alongside soluble phenolics distinguishes it from other plant-based functional ingredients. Soluble phenolics are rapidly absorbed in the upper gastrointestinal tract and exert early systemic antioxidant and anti-inflammatory effects. In contrast, insoluble-bound phenolics, closely associated with the fiber matrix, resist digestion and reach the colon intact, where microbial fermentation gradually releases them into bioactive metabolites [24,31,32]. This is consistent with our observed improvements in gut barrier function, reflected in reduced plasma FITC-dextran and LPS levels, preservation of tight junction protein ZO-1, as well as restored colon length and mitigated histopathological damage. Additionally, beyond of the modulation of gut permeability, RWGP significantly reduced circulating IL-1β levels, reinforcing its anti-inflammatory profile. RWGP did not significantly reduce systemic lipid peroxidation (TBARS), which may reflect a primarily localized action in the gut or delayed systemic modulation via microbial metabolites [33,34,35,36]. Comparable matrix effects have been observed in cocoa husk and apple pomace, where fiber–phenol complexes promoted gut health and immune regulation [37,38]. These findings are consistent with our prior observation that RWGP’s cardioprotective effects are not reproduced by fiber supplementation alone, supporting the notion that RWGP’s health effects stem from the concerted activity of its diverse phytochemical composition rather than from isolated compounds [19]. Notably, statin treatment—despite reducing systemic oxidative and inflammatory markers as well as increasing survival—did not replicate RWGP’s benefits on gut structure or permeability, suggesting that the full RWGP matrix—including phenolics, flavonoids, and fiber—could support cardiovascular outcomes beyond lowering lipid levels [39,40,41,42].

In addition to total phenolics, RWGP contains substantial levels of flavonoids, particularly catechins and procyanidins, which are nearly equivalent in content and may exert complementary effects. Phenolic acids and flavonoids share antioxidant and anti-inflammatory properties, including the inhibition of lipid peroxidation, modulation of NF-κB signaling, and suppression of pro-inflammatory cytokine production [43,44,45,46,47,48]. Their co-occurrence within RWGP’s matrix suggests a synergistic mechanism whereby both classes contribute to enhanced gut barrier integrity and immune modulation, where they could act locally before systemic absorption [49]. Flavonoids such as catechins and procyanidins—which are present in both free and bound forms in RWGP—are partially absorbed in the upper gastrointestinal tract while most are catabolized by colonic microflora where they can modulate oxidative stress, inflammation, and gut epithelial responses [50,51]. Together, these compounds shape gut barrier integrity through distinct yet overlapping mechanisms, reinforcing the relevance of the whole matrix in supporting intestinal and systemic health [38,52]. However, future studies should aim to delineate the relative contribution of phenolics and flavonoids—both in free and bound forms—to better understand their individual and combined roles in shaping gut–host interactions and downstream cardiometabolic outcomes.

While our findings suggest that the beneficial effects of RWGP on gut barrier integrity and systemic inflammation may be mediated in part through interactions with the gut microbiota, we did not directly assess microbial composition, fermentation products, or related physiological indicators such as cecum weight or pH [53]. Notably, grape pomace has been shown to shape the intestinal milieu in ways that extend beyond short-chain fatty acid (SCFA) production, influencing the broader microbial metabolome and its systemic impact [54]. Given that the release and bioactivity of bound phenolics are largely dependent on microbial fermentation, future studies should incorporate microbiota profiling (16S rRNA sequencing), quantification of SCFAs (butyrate, acetate, propionate), and additional gut fermentation markers to clarify the mechanistic link between RWGP intake, microbial metabolism, and host cardiometabolic health [55]. Notably, other microbial-derived metabolites, such as phenylacetic and benzoic acid derivatives, secondary bile acids, aromatic amino acid metabolites, neurotransmitters, and B vitamins, have been increasingly recognized as mediators of host cardiometabolic health, including immune signaling, lipid metabolism, vascular tone, and redox balance [56]. Moreover, the dietary fiber in RWGP not only serves as a carrier for insoluble-bound phenolics but also likely modulates their fermentation kinetics and microbial accessibility [20]. Therefore, the exploration on structural properties of RWGP fiber (e.g., glycosidic linkages, polymerization degree) will provide essential insights into how the fiber–phenolic matrix of RWGP shapes gut microbial ecology and its downstream effects on systemic inflammation and cardiovascular protection. Another limitation of our study is the relatively small sample size, which, although sufficient to detect significant effects in several endpoints, may limit the generalizability of the findings; in this way, future studies involving larger cohorts are warranted to confirm and extend these observations. These efforts would also support the sustainable valorization of winery residues and align with circular economy principles in the agri-food sector [57,58].

## 5. Conclusions

Our findings demonstrate that the cardioprotective and gut-modulating effects of RWGP are driven by the synergistic interactions among its phenolics, flavonoids, and dietary fiber, rather than any single component. This integrated matrix supports gut barrier integrity, attenuates systemic inflammation, and improves survival in diet-induced ischemic heart disease. These results highlight the therapeutic potential of RWGP as a sustainable functional food and underscore the importance of evaluating whole-food matrices in chronic disease prevention. Future work should further elucidate matrix–microbiota interactions, validate efficacy in clinical settings, and optimize RWGP’s formulation for functional applications within circular economy frameworks.

## Figures and Tables

**Table 1:** HPLC-DAD step gradient. Elution time ranges for mobile phase step gradient in HPLC-DAD.

Time (min)	Acetonitrile (%)	Acidified Water (%)
0–30	15	85
30.1–45	25	75
45.1–55	40	60
55.1–60	50	50
60.1–65	80	20
65.1–75	15	85

**Table 2:** Total phenolic and flavonoid content of the soluble (free, esterified, etherified) and insoluble fractions of RWGP flour.

Fraction	Total Phenolics	Total Flavonoids
	(mg GAE/100 g)	(mg QE/100 g)
Free	509 ± ${43\;}^{b}$	535 ± ${6.7\;}^{b}$
Esterified	270 ± ${20\;}^{c}$	116 ± ${6.9\;}^{c}$
Etherified	75 ± ${9\;}^{d}$	49 ± ${5.9\;}^{c}$
Insoluble-bound	830 ± ${42\;}^{a}$	656 ± ${56\;}^{a}$
Total	1684	1356

Values are presented as mean ± SD (n = 3). Different letters within the same columns (a–d) indicate significant differences at *p* < 0.05. RWGP: Red wine grape pomace. GAE: Gallic acid equivalent. QE: Quercetin equivalent.

**Table 3:** Phenolic acids and flavonoids in soluble (free, esterified, and etherified) and insoluble-bound fractions of RWGP flour.

Compound	Free	Esterified	Etherified	Insoluble
	(mg/100 g)	(mg/100 g)	(mg/100 g)	(mg/100 g)
Gallic acid	9.95 ± ${0.03\;}^{c}$	17.05 ± ${0.22\;}^{b}$	5.73 ± ${0.04\;}^{d}$	72.38 ± ${0.03\;}^{a}$
p-Coumaric acid	nd	21.20 ± ${0.20\;}^{a}$	nd	34.91 ± ${0.57\;}^{b}$
Catechin	11.82 ± ${0.21\;}^{b}$	5.17 ± ${0.20\;}^{c}$	0.25 ± ${0.03\;}^{d}$	26.65 ± ${1.79\;}^{a}$
Epicatechin	40.75 ± ${0.68\;}^{a}$	tr	0.33 ± ${0.25\;}^{c}$	8.00 ± ${1.34\;}^{b}$
Epigallocatechin gallate	0.21 ± 0.01	nd	nd	nd
Procyanidin B2	nd	1.16 ± ${1.01\;}^{a}$	nd	7.19 ± ${0.11\;}^{b}$
Kaempferol	2.16 ± 0.07	nd	nd	nd

Values are presented as mean ± SD (n = 3). Overall *p*-values were obtained using the one-way ANOVA followed by Tukey’s post hoc test. Different letters within the same row (a–d) indicate significant differences at *p* < 0.05. nd is not detected, tr: trace.

**Table 4:** Dietary intake and body weight parameters.

	CD	HFCD-CD	HFCD-ST	HFCD-RWGP
Dietary intake (g/day)	4.30 (3.60–4.70${)\;}^{a}$	3.70 (3.30–4.30${)\;}^{a}$	3.85 (3.60–5.20${)\;}^{a}$	4.60 (3.60–5.30${)\;}^{a}$
Fat intake (g/day)	0.24 (0.20–0.26${)\;}^{b}$	0.54 (0.48–0.62${)\;}^{a}$	0.56 (0.52–0.75${)\;}^{a}$	0.69 (0.54–0.80${)\;}^{a}$
Fiber intake (g/day)	0.66 (0.55–0.72${)\;}^{\mathrm{ab}}$	0.56 (0.50–0.65${)\;}^{b}$	0.58 (0.54–0.78${)\;}^{b}$	1.00 (0.80–1.20${)\;}^{a}$
Water intake (mL/day)	5.70 (5.40–6.20${)\;}^{a}$	6.4 (5.10–9.90${)\;}^{a}$	5.50 (4.80–6.20${)\;}^{a}$	4.20 (3.90–8.70${)\;}^{a}$
Starting body weight (g)	28.2 (25.3–29.9${)\;}^{a}$	27.1 (26.4–30.1${)\;}^{a}$	27.1 (25.3–27.7${)\;}^{a}$	26.60 (25.6–27.7${)\;}^{a}$
Final body weight (g)	30.1 (28.7–31.3${)\;}^{a}$	23.80 (22.8–25.0${)\;}^{b}$	27.2 (26.5–28.1${)\;}^{\mathrm{ab}}$	27.2 (25.6–29.2${)\;}^{\mathrm{ab}}$
Body weight gain (g)	1.9 (0.2–3.4${)\;}^{a}$	−3.9 (−5.1– 0.0${)\;}^{b}$	0.1 (−1.2–2.8${)\;}^{\mathrm{ab}}$	1.3 (−2.1–2.5${)\;}^{\mathrm{ab}}$

Calculations based on a previous nutritional analysis [19]. Values are expressed as median (interquartile range), n = 6. Kruskal–Wallis test, followed by Dunn’s test for multiple comparisons; Different letters in the same row (a, b) represent a significant difference at *p* < 0.05.

## Data Availability

Data is contained within the article.
